# Surgically-oriented anatomical study of mandibular premolars: A CBCT study

**DOI:** 10.4317/jced.55848

**Published:** 2019-10-01

**Authors:** Stefano Corbella, Martino Baruffaldi, Isabella Perondi, Silvio Taschieri

**Affiliations:** 1 Visiting Professor, Department of Biomedical, Surgical and Dental Sciences, Università degli Studi di Milano, Milan, Italy. IRCCS Istituto Ortopedico Galeazzi, Dental Clinic, Milan, Italy. Professor, Institute of Dentistry, Dept. of Oral Surgery, I. M. Sechenov First Moscow state medical University, Moscow, Russia; 2 Department of Biomedical, Surgical and Dental Sciences, Università degli Studi di Milano, Milan, Italy. IRCCS Istituto Ortopedico Galeazzi, Dental Clinic, Milan, Italy

## Abstract

**Background:**

The knowledge of root canal anatomy and of the anatomical relationship should be considered mandatory when planning surgical endodontics. The aim of the study was to investigate the anatomical features of mandibular premolars, evaluating their relationship with mental nerve.

**Material and Methods:**

CBCT scans were evaluated recording the number of roots, root canal configuration and the relationship with mental nerve of 100 mandibular premolars. After simulating a resection of 3 mm of the root, the shape and the number of canals, and the distance to the buccal and lingual bone plate and to the mental foramen was evaluated.

**Results:**

The one root - one canal configuration was the most common configuration. The mental foramen was located at the level of MSPs in 40% cases, and it was between MSP and MFP in 46% of cases. The distance between the apex and the vestibular plate was lower than the distance to the lingual one.

**Conclusions:**

We found a significant heterogeneity in the anatomy of mandibular premolars. CBCT could be considered important when planning surgical endodontics in this region.

** Key words:**Cone-Beam Computed Tomography, bicuspid, root canal, root canal therapy, apicoectomy, mandibular nerve.

## Introduction

The objective of non-surgical endodontic treatment is to achieve a complete removal of the pulpal tissue or debris, to obtain an adequate disinfection of the root canal system, and to perform a tridimensional filling of the root canal space by means of a biocompatible obturation material (1-3). Many factors can influence the prognosis of endodontically treated teeth: the presence of a preoperative periapical lesion (4,5), the radiographic evidence of voids in the tridimensional filling as evaluated through periapical radiographs (5), an inadequate coronal restoration (5), and the extension of root obturation in relation to the position of the apex (4,6). One of the most common condition, predisposing to endodontic failures even in cases of adequate treatment, is the missing anatomy that is related to the presence of anatomical abnormalities, lateral canals, and to complex root canal anatomy (7).

Surgical endodontics could be the treatment of choice in those cases of failed endodontic treatment, where a nonsurgical approach is not feasible, or the possibility to improve the previous result without surgery was considered improbable (8). Since the presence or persistence of peri-radicular disease can be observed in more than 30% of all root-filled teeth (9-11), surgical endodontics could be the treatment of choice in many cases.

The knowledge of the anatomical characteristics of the tooth and of its relationship with the neighboring anatomical structures is mandatory to perform an effective and safe surgical endodontics treatment (12,13). Moreover, the use of magnification devices, such as surgical microscope and surgical loupes, can provide a significant assistance in the identification of anatomical details during the surgical procedure, thus reducing the possibility to create injuries to teeth-surrounding structures (12).

Many authors found that mandibular premolars are frequently associated with the presence of anatomical variations that can have, on the basis of the previously described assumptions, an impact on the outcome of endodontic treatment, both nonsurgical and surgical (14-17). As compared to other teeth, premolars showed high heterogeneity in terms of anatomical features (18) that was related to several factors including age, sex, and ethnicity (14-16). The anatomical variations are more frequent in mandibular first premolars (MFPs), having up to four roots and variable anatomy of the root canal system, than in mandibular second premolars (MSPs), usually presenting with one single root and one single canal (14,15,19).

Since when it was introduced in dental radiology in order to lower the dose of radiation required for tri-dimensional imaging, Cone Beam Computed Tomography (CBCT) has become a useful device also in Endodontics practice (20,21). The guidelines for the use of CBCT in endodontic practice were discussed in two position papers published by the American Academy of Endodontics (22) and European Society of Endodontics (23). Both papers agreed that in the presence of complex teeth anatomies, that could not adequately be identified by bi-dimensional radiography, CBCT could be used to investigate anatomical features, particularly when surgery is under consideration (22,23). Regarding mandibular cases, CBCT was an important tool for the study of the course of mandibular nerve and its relationship with the apex of the teeth; this relationship could be of great importance in planning and performing surgical endodontics (24).

The aim of the present study was to investigate the anatomical features of mandibular premolars, evaluating the relationship with mandibular nerve and with mental foramen, simulating root end resection.

## Material and Methods

All the phases of the study were conducted in adherence to the Helsinki Declaration and to its further modifications (25).

The present investigation was a retrospective noninterventional study on CBCT scans that were taken for purposes related to oral surgery interventions, such as implant positioning, mandibular third molar surgery, cyst enucleation, and for diagnostic purposes. All patients provided their written informed consent before the scan, also providing their consent to use the content of the scans for further research.

The CBCT scans were taken with Orthophos SL 3D (Dentsply Sirona, Wals bei Salzburg, Austria) set at 5000ms, 7mA, 85kV, with a resolution of 0.08 mm; the images were examined with the software provided with the CBCT machine (Sidexis 4, Dentsply Sirona, Wals bei Salzburg, Austria).

-Eligibility criteria

The radiographic records were selected consecutively and evaluated for the inclusion in the study on the basis of the following eligibility criteria:

i) the subject’s age was equal to, or more than, 18 years old and the subject was able to understand and sign an informed consent form; ii) being of good quality, clearly identifying the features that are the object of the study; iii) being mandibular scans, showing both the MFP and MSP on both sides; iv) the mandibular permanent premolars were not endodontically treated; v) not having any condition potentially limiting the possibility to identify the structures that are object of the study, such as neoplasms, cysts, large peri-radicular lesions, artifacts, internal or external root resorption, and pulpal calcification.

On the basis of the previously listed criteria, we selected a total of 50 mandibular scans, accounting for 100 MFPs and 100 MSPs.

-Outcomes and data extraction

The primary outcome of the study was to identify the roots and root canal anatomy of mandibular permanent premolars in relation to surgical endodontics procedures. The number of roots was recorded and root canal system anatomy was evaluated on the basis of the classification proposed by Vertucci in 1978: (17) Type I) 1-1 configuration (one canal and one apical foramen); Type II) 2-1 configuration; Type III) 1-2-1 configuration; Type IV) 2-2 configuration; Type V) 1-2 configuration; Type VI) 2-1-2 configuration; Type VII) 1-2-1-2 configuration; Type VIII) 3-3 configuration.

Other anatomical characteristics were recorded: i) the number of apices; ii) the presence of a lateral canal; iii) the presence of a C-shape anatomy.

In order to simulate the execution of a 90 degrees root-end resection in a surgical endodontics procedure several parameters were measured at a resection level located 3 mm coronal to the anatomical apex, according to the surgical recommendations of the “modern technique” (12)): i) number of root canals present at that level; ii) shape of the canal(s) (round or oval); iii) presence of an isthmus; iv) size of the canal(s); v) distance in mm between the surgical apex and the vestibular / lingual cortical plate; vi) distance in mm between the surgical apex and the mental foramen, measured both in mesio-distal direction and in bucco-lingual direction; vii) the position of the mental foramen in relation to mandibular premolars (0: apical to the MSP; 1: apical to the MFP; 2: between MSP and MFP; 3: distal to MSP; 4: mesial to MFP).

All the measures were obtained by the authors, having more than five years of experience in the interpretation of tridimensional radiographic images. They were calibrated before the beginning of the study by viewing 10 sample scans and analyzing them jointly.

-Data analysis

The statistical analysis of data was performed by one operator by using a dedicated software (IBM SPSS Statistics Version 22, IBM, Armonk, NY, USA).

Descriptive statistics was provided by presenting means and standard deviations for continuous data and frequencies for categorical variables. For continuous normally distributed variables differences between groups were calculated by using Student’s T test. The linear and logistic regression method was used for each study variable (independent variable) in relation to any primary outcome (dependent variable). The level of significance was set *P*=.05.

## Results

**Figure 1:**
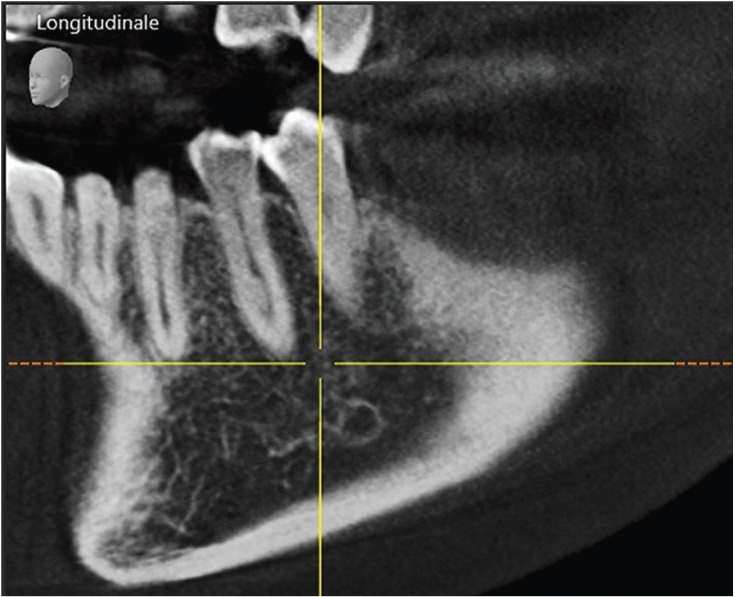
Example of one examined MFP with particular root canal configuration.

The full mandibular CBCT scans belonged to 25 men and 25 women, all Caucasians born in Italy, aging 44.5 ± 17.2 years (ranging from 18 to 83 years). As for root number, 95% of MFPs and 97% of MSPs had one single root. The frequencies of root canal anatomy configurations are presented in  Table 1 (Fig. 1). Evaluating sections 3-mm coronal to the anatomical apex we found two distinct canals in 18% of MFPs and in 7% of the MSPs, having the others one single canal. Regarding the shape of the canal at 3-mm level, it was round-shaped in 67% of MFPs and in 73% of MSPs, oval-shaped in 15% of MFPs and 20% of MSPs, one oval-shaped and one round-shaped in 9% of MFPs and 7% of MSPs and both round-shaped in 9% of MFPs. An isthmus could be found in 4% of MFPs and in none of the MSPs.

**Table 1:**
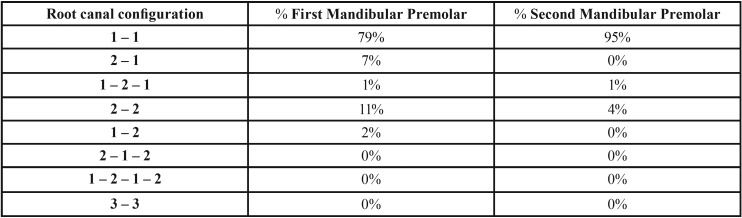
Distribution (%) of root canal configurations.

The mean diameter of the canal, at the resection level, was 0.38 ± 0.29 mm in the MFPs and 0.41± 0.35 mm in the MSPs; when a second canal was present its diameter was 0.19 ± 0.11 mm in the MFPs 0.18 ± 0.11 mm in the MSPs. For both teeth the second canal, when present, was significantly smaller than the main one which was located in a vestibular position (*P*<0.0001).

**Figure 2:**
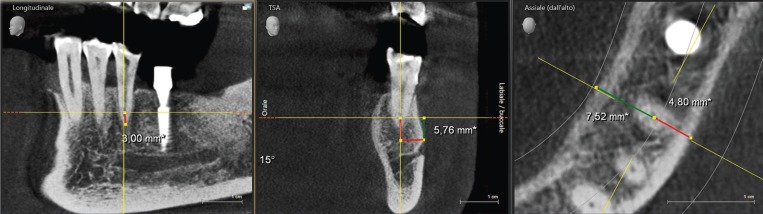
Example of the measuring process. Note the clear visibility of all anatomical features.

Distances between anatomical structures and the resection level are presented in  Table 2. In Figure 2, it is shown one of the cases included and measured. The mental foramen was located (evaluating its mesio-distal position) apical to the MSPs in 40% of cases, apical to the MFPs in 10% of cases, between MFPs and MSPs in 46% of cases, and distal to the MSPs in 4% of cases.

**Table 2:**
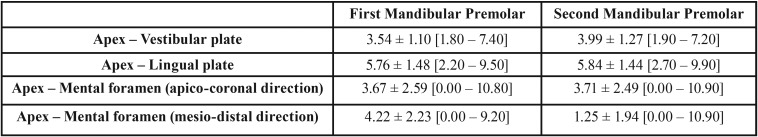
Distances (in mm) between the surgical apex and the surrounding anatomical structures.

## Discussion

In the present study we found a significant heterogeneity in permanent maxillary premolars anatomy and their relationship with mental foramen. Such heterogeneity could be considered as one potential risk factor for failures of endodontic treatment, both surgical and nonsurgical.

Certain factors have to be examined to weight the external validity of the obtained results. Firstly, since some papers found differences in anatomical features of teeth from different ethnic groups (19), the inclusion of only Caucasians (Italians) could be a potential limit to the generalization of the results to other populations. Secondly, the use CBCT scans, even if validated for the use in Endodontics, could not be compared to in vitro studies on extracted tooth, that allow a direct visualization of the anatomy. In this particular study, we excluded scans showing artifacts and other images that could potentially influence the visualization of the anatomical characteristics; however, the presence of root canal calcifications could have confounded the results; however we can consider this a rare occurrence (26). Another limitation could be represented by the relatively small sample size. However, we should consider that, as reported in one systematic review of the literature, a similar size of the sample was studied in other published papers (19). Finally, we should consider that the choice of the voxel size of 0.08 mm could have influenced the results in terms of inability of detecting structures smaller than this size.

In the present study, we found that MFPs and MSPs showed two roots in 5% and 3% of cases, respectively. Cleghorn in the review published in 2007, reported that a second root could be found in 3.9% (27) - 15% (28) of MFPs, depending on the study that was considered (15). Considering different ethnic groups, 5.4% of MFPs belonging to African Americans and 16.2% of those belonging to Caucasians had second root, as evaluated in one study (16). Other recent papers about the anatomy of MFPs reported the presence of a second root in 1% of cases in a Taiwanese population (29) and in 3.6% of cases in a Turkish cohort (30). As for MFPs, the prevalence of a second root in the present study was comparable to the one presented by Trope in 1986 (5.5% in the Caucasian Americans group) (16) and by Geider in 1989 (6.4% in Caucasian Europeans), both exploring ethnic groups that were similar to the one in our study (31). With regard to MSPs, in literature, the presence of a second root was rare (less than 1%) while we found it in 3% of teeth (14,19).

The most common root canal system configuration found was Vertucci Type I (1-1) accounting for 79% and 95% for MFPs and MSPs respectively; such proportions were consistent with those reported in literature, despite a significant heterogeneity among studies. Kottoor and coworkers found this configuration in 72.2% (weighted mean among all included studies) of MFPs and 83.6% of mandibular second premolars (97.5% of teeth in Caucasians). It can be observed that probably ethnicity plays an important role in determining root canal configuration. In our study, MFPs Type IV (2-2) configuration was the second most common, and this was confirmed also by the study by Kottoor and coworkers that reported 10.7% of cases showing this configuration (19). The same was found for the MSPs, showing Type IV configuration in 4% of cases in our study and 3.6% of cases in one systematic review of the literature (19). Moreover, our results appeared to be comparable to those published by Pedemonte and coworkers in 2018, regarding a population made of Belgian and Chilean people (32). Similar prevalence of Type I and Type V root canal configurations was also found in the paper published by Zhang and colleagues in 2017 (China) (33). Interestingly, the data published by Burklein and coworkers in 2017 reported that Type V configuration was the most prevalent among MFPs and MSPs, being this data significantly different from those reported in the present study (34). It could be hypothesized that such difference was due to the methods of detecting a second apical foramen in CBCT scans.

The concept of “modern technique” in Endodontic microsurgery included the need of minimal access to the apical structure by means of microsurgical techniques and magnification devices (12). The knowledge and awareness of the anatomical structures is fundamental to minimize the size of the ostectomy, thus reducing the risk of injuries of the neurovascular bundles (12). Moreover, the knowledge of the shape and of the number of apices to be sealed after root-end resection should be considered an important factor to minimize the risk of apical leakage, since it was found that apices with an oval shape were more prone to this complication than round ones (35). It is notable that approximately two third of the teeth have one single round apex.

According to other studies on the same topic (36,37), we evaluated the distance between the surgical site and the surrounding anatomical structures, making a virtual root-end resection of 3-mm length. In the majority of cases the apical foramen was located at the level of the MSP or between the two premolars (38,39). The distance between the surgical site and the mental foramen was similar to the measure of the resection (3 mm) and this could be a major factor in supporting the use of tridimensional radiographic imaging (CBCT) before surgical endodontics in this area. Considering mean values, the mental foramen was significantly closer to the MSP than to MFP, and this was a partial confirmation of the results by Wu and coworkers (35). However, due to the significant variability (range) of the values, a careful preoperative evaluation is mandatory in order to locate the exact position of the neurovascular mandibular bundle and mental foramen, particularly in this area (40).

The estimation of the depth of the ostectomy could be important when planning surgical endodontics, to minimize the invasiveness of the procedure. One recent study reported the need of approximately 4.0 – 4.5 mm of resection when performing surgical endodontics at the level of the premolars, being bucco-lingual size of the roots of at least 3.47 – 3.39 mm in the region of MFPs and 3.36 – 3.44 mm in the region of MSPs and a buccal bone width of 0.85 – 0.88 mm in the region of MFPs and of 1.17 – 1.30 mm in the region of MSPs (37). In our study we measured the distance between the center of the canal or the mid-point between two canals were present and the buccal external plate; due to this reason the results could not be compared to those from the study by Zahedi and coworkers (37). However, we found that the midpoint of the canal could be found approximately between, on average, 3 and 4 mm from the buccal plate, taking in consideration a significant variability. Similar findings were reported in other papers with the aim of measuring the width of the buccal plate of the mandible (41,42).

## Conclusions

Despite the limitations of the study, mandibular premolars showed a substantial variability of their root canal anatomy and this aspect should be taken in consideration during the planning of surgical intervention. Furthermore, the relationship between MFPs and MSPs and the surrounding anatomical structures should be carefully evaluated when planning surgical endodontics in that area, and CBCT is a valuable instrument for the visualization of anatomical features of these teeth, particularly in all cases when anatomy was not clearly identifiable by bi-dimensional imaging.
